# Dyslipidemia among Patients with Ischemic Stroke in the Department of Medicine of a Tertiary Care Centre: A Descriptive Cross-sectional Study

**DOI:** 10.31729/jnma.7491

**Published:** 2022-06-30

**Authors:** Sushil Baral, Asmita Pokhrel, Shyam Kumar B.K., Rupesh Kshetri, Prashant Regmi, Prajwal Gyawali

**Affiliations:** 1 Department of Internal Medicine, National Academy of Medical Science, Bir Hospital, Mahabouddha, Kathmandu, Nepal; 2 Department of Biochemistry, Nepal Medical College and Teaching Hospital, Jorpati, Kathmandu, Nepal; 3 Nepalgunj Medical College, Chisapani, Banke, Nepalgunj, Nepal; 4 University of Iowa Hospitals and Clinics, Iowa City, USA; 5 Adelaide Medical School, The University of Adelaide, Adelaide, Australia; 6 School of Health and Medical Sciences, University of Southern Queensland, Queensland, Australia

**Keywords:** *dyslipidemia*, *ischemic stroke*, *lipid*, *prevalence*

## Abstract

**Introduction:**

Stroke is a leading cause of morbidity and disability in Asian population. Dyslipidemia is considered a major risk factor for various cardiovascular diseases. The study aimed to find the prevalence of dyslipidemia among patients with ischemic stroke in the Department of Medicine of a tertiary care centre.

**Methods:**

This is a descriptive cross-sectional study conducted among 150 diagnosed cases of ischemic stroke admitted in the Department of Medicine from 1^st^ October, 2020 to 1^st^ October, 2021. The ethical clearance was taken from the Institutional Review Committee (Reference number: 358/2077/78). Fasting blood samples were collected from the patients, serum lipids were measured and atherogenic indices of plasma were calculated. Demographic, anthropometric and cardiovascular risk factors related data were collected. Data were entered in Microsoft Excel 2010 and analysis was using the Statistical Package for the Social Sciences version 22.0. Point estimate at 95% Confidence Interval was calculated along with frequency and proportion for binary data, and mean and standard deviation for continuous data.

**Results:**

The prevalence of dyslipidemia among the ischemic stroke patients was 120 (80.00%) (73.6086.40 at 95% Confidence Interval). High total cholesterol was found in 64 (53.33%) patients, high triglycerides in 70 (58.33%), high low-density lipoprotein cholesterol in 54 (45.00%) and low high-density lipoprotein cholesterol in 51 (42.50%) patients.

**Conclusions:**

The prevalence of dyslipidemia among ischemic stroke patients was higher than the studies done in similar settings.

## INTRODUCTION

Stroke is a leading cause of morbidity and disability in Asian population.^1^ About 80% of strokes are focal cerebral ischemia due to arterial occlusion and 20% by hemorrhages.^2^ Population aged 60 or over is rising faster than young age group worldwide.^3,4^ Reports have suggested that about 80% strokes occurred in individuals aged >65 years, among which 50% were aged ≥70 years and nearly 25% aged >85 years.^5^ However one third of strokes occur in persons younger than 65 years.^6^

Dyslipidemia is a known cardiovascular risk where association become more apparent in both sexes.^7^ A study done in Nepal showed a three-fold rise in the incidence of dyslipidemia in stroke patients. ^8^ Though, the incidence of stroke in both sex is not completely elucidated. Several risk factors such as dyslipidemia, hypertension, diabetes mellitus has been recognized in stroke outcome.^9^

The study aimed to find the prevalence of dyslipidemia among patients with ischemic stroke in the Department of Medicine of a tertiary care centre.

## METHODS

A descriptive cross-sectional study was carried out in the Department of Medicine at National Academy of Medical Sciences (NAMS), Bir Hospital, Kathmandu, Nepal. The diagnosed cases of ischemic stroke in the hospital from 1^st^ October, 2020 to 1^st^ October, 2021 were recruited in the study. The ethical clearance was taken from the Institutional Review Committee, NAMS (Reference number: 358/2077/78). This study included all the patients with first stroke diagnosed by development of sudden onset neurological deficit affecting a vascular territory with sustained deficit for more than 24 hours with the evidence of stroke on Magnetic Resonance Imaging (MRI) or Non-contrast Computed Tomography (NCCT) scan confirmed by a neurologist. Age, sex, socioeconomic status, demographic, history of hypertension, diabetes, history of smoking and alcohol consumption were recorded. The patients without neuroimaging were excluded. Convenience sampling was done.

The sample size was calculated using the following formula:


$$\begin{array}{ll} \text{n} & ={\text{Z}}^{2}\times \frac{\text{p}\times \text{q}}{{\text{e}}^{\text{2}}}\\ & =1.96^{2}\times \frac{0.5\times 0.5}{0.08^{2}}\\ & =150 \end{array}$$

Where,

n = minimum required sample size
Z = 1.96 at 95% Confidence Interval (CI)
p = prevalence taken as 50% for maximum sample size calculation
e = margin of error, 8%

Hence, a sample size of 150 was taken.

Overnight fasting blood samples (8-12 hour fasting) were collected from the patients and centrifuged to separate serum. Lipid profiles (TC-Total Cholesterol, HDL-c-High Density Lipoproteins-cholesterol, TG-Triglyceride) were measured using enzymatic methods. Low Density lipoproteins-cholesterol (LDL-c) was calculated by using Fridewald's formula.^10^

Dyslipidemia is defined according to the Third Report of the National Cholesterol Education Program (NCEP) Adult Treatment Panel III.^11^

Non-HDL-c was calculated as the difference between TC-HDL-c. Atherogenic Indices of Plasma (AIP) was calculated as AIP= Log (TG/serum HDL-c). As per a study, AIP values were interpreted where AIP values -0.3 to 0.1 were considered as low risk, 0.10 to 0.24 as medium risk, and above 0.24 as high risk for cardiovascular disease.^12^ Castelli Risk Index (CRI) I and II were calculated as: CRI-I = Serum TC/Serum HDL-c, CR-I II = Serum LDL-c /Serum HDLc. Atherogenic Coefficient (AC) was calculated as: (Serum TC-Serum HDL-c)/HDL-c.

The data were entered in Microsoft Excel 2010. For data analysis, Statistical Package for the Social Sciences (SPSS) version 22.0. was used. Point estimate at 95% CI was calculated along with frequency and proportion for binary data along with mean and standard deviation for conitnuous data.

## RESULTS

The prevalence of dyslipidemia among the ischemic stroke patients in this study was 120 (80.00%) (73.6086.40 at 95% Confidence Interval). High total cholesterol was found in 64 (53.33%) patients with mean lipid level of 198.83±53.74, Similarly, high triglycerides in 70 (58.33%) patients with mean triglyceride value of 161.81 ± 62.09. Likewise high LDL cholesterol in 54 (45.00%) with mean LDL 124.34±50.53, and low HDL cholesterol was found in 51 (42.50%) patients with mean HDL 42.12±7.61 (Table 1).

**Table 1:** Lipid profile in different age groups (n = 120).

Lipid profile	<50 years n (%)	>50 years n (%)	Total n (%)
High total cholesterol	22 (57.89)	42 (51.22)	64 (53.33)
High triglyceride level	22 (57.89)	48 (58.53)	70 (58.33)
Low HDL-c	16 (42.10)	35 (42.68)	51 (42.50)
High LDL-c	20 (52.63)	34 (41.46)	54 (45.00)

Gender-wise distribution showed that the stroke patients with dyslipidemia included 72 (60.00%) males and 48 (40.00%) females. Similarly, 40 (55.56%) males and 24 (50.00%) females had high TC while 37 (51.38%) males and 33 (68.75%) females had high TG. Likewise, 33 (45.83%) males and 21 (43.75%) females had high LDL while 34 (47.22%) males and 17 (35.41%) females had low HDL. The serum mean values of Total Cholesterol (TC), High Density Lipoprotein-cholesterol (HDL-c), TG and AIP were found to be higher among females (Table 2).

**Table 2:** Mean serum lipids and atherogenic factors in patients with ischemic stroke (n = 120).

Parameters	Male Mean±SD	Female Mean±SD	Total Mean±SD
TC (mg/dl)	198.10±57.01	199.92±49.00	198.83±53.74
TG (mg/dl)	158.59±70.30	166.64±47.49	161.81±62.09
HDL-c (mg/dl)	40.83±7.95	44.02±6.70	42.12±7.61
LDL-c (mg/dl)	125.54±51.55	122.54±49.43	124.34±50.53
Non-HDL-c (mg/dl)	157.26±53.12	155.87±49.48	156.70±51.49
TC/HDL-c	4.88±1.21	4.64±1.47	4.79±1.32
LDL/HDL-c	3.08±1.15	2.88±1.40	3.00±1.25
TG/HDL-c	4.02±1.94	3.84±1.17	3.95±1.67
AIP	0.55±0.20	0.56±0.13	0.55±0.18
AC	3.88±1.21	3.64±1.47	3.79±1.32

The mean age of the patients with ischemic stroke presenting with dyslipidemia was 59.98±13.50 years. Among them, 72 (60.00%) were male and 48 (40.00%) were female. In this study, 82 (68.33%) were above 50 years (elderly) whereas 38 (31.67%) were below 50 years (non-elderly). In our study, 87 (72.50%) patients belonged to Hindu religion and 58 (48.3%) patients were farmers by occupation (Table 3).

**Table 3:** Baseline characteristics of ischemic stroke patients with dyslipidemia (n = 120).

Variables	n (%)
Age (Mean ±SD) (in years)	59.98±13.50
**Sex**	
Male	72 (60.00)
Female	48 (40.00)
**Age-group (years)**	
29-50	38 (31.67)
50-60	20 (16.67)
60-70	30 (25.00)
70-82	32 (26.67)
**Religion**	
Hindu	87 (72.50)
Buddhist	21 (17.50)
Christian	11 (9.67)
Muslim	1 (0.83)
**Occupation**	
Farmers	58 (48.33)
Businessman	26 (21.67)
Housewife	22 (18.33)
Office workers	13 (10.83)
Other	1 (0.83)

In our study 112 (93.33%) of patients presented with hemiparesis, 25 (20.83%) presented with slurring of speech and aphasia, 17 (14.17%) presented with headache, 17 (14.17%) presented with vomiting, 6 (5.00%) presented with sensory disturbances, 5 (4.17%) presented with loss of consciousness, and 2 (1.67%) presented with convulsion (Table 4).

**Table 4:** Clinical presentation in ischemic stroke with dyslipidemia (n = 120).

Symptoms	Male (n= 72) n (%)	Female (n= 48) n (%)	Total (n= 120) n (%)
Hemiparesis	71 (98.61)	41 (85.42)	112 (93.33)
Slurring of speech and aphasia	15 (20.83)	10 (20.83)	25 (20.83)
Headache	7 (9.72)	10 (20.83)	17 (14.17)
Vomiting	8 (11.11	9 (18.75)	17 (14.17)
Sensory disturbances	1 (1.39)	5 (10.42)	6 (5.00)
Loss of consciousness	3 (4.17)	2 (4.17)	5 (4.17)
Convulsion	1 (1.39)	1 (2.08)	2 (1.67)

In this study, the commonest risk factor for the strokewas hypertension which was present in 74 (61.67%) o1patients. Then followed by 70 (58.33%) were currentsmokers and65 (54.17%)were alcoholconsumers. Diabetes waspresent in 34 (28.33%),6 (5.00%)patients had family history of stroke and 7 (5.83%)patients had atrial fibrillation (Table 5).

**Table 5:** Factors in ischemic stroke patients with dyslipidemia (n = 120).

Factors	Male n (%)	Female n (%)	Total n (%)
Hypertension	46 (63.89)	28 (58.33)	74 (61.67)
Smoker	49 (68.05)	21 (43.75)	70 (58.33)
Alcohol	43 (59.72)	22 (45.83)	65 (54.17)
Type 2 diabetes mellitus	19 (26.39)	15 (31.25)	34 (28.33)
Atrial fibrillation	4 (5.56)	3 (6.25)	7 (5.83)
Family history of stroke	2 (2.78)	4 (8.33)	6 (5.00)

## DISCUSSION

The prevalence of dyslipidemia among ischemic stroke patients in this study was 80% with hypertriglyceridemia being the most common finding which consisted of 58.3%. This finding was supported by study done in our country which show that dyslipidemia was present in 77.2%.^4^

Similarly, a study done in Nigeria showed an incidence of dyslipidemia was 92.30% which is significantly higher as compared to our study.^13^ This may support that, a study done in our country states that the high dominance of dyslipidemia could be due to the sedentary lifestyle and environment changes among Nepalese patients and found a three-fold rise in the incidence of dyslipidemia in stroke patients.^8^

High total cholesterol was found in 53.33% patients with mean 198.83 ± 53.74, high triglycerides in 58.33% patient with mean 161.81±62.09, high LDL cholesterol in 45% with mean 124.34 ± 50.53, and low HDL cholesterol was found in 42.5% patients with mean 42.12 ±7.61. A study done showed the mean of TC, TG, LDL-c higher than our finding. This study also said that dyslipidemia involves the production of atherosclerosis through increasing of plasma cholesterol, triglycerides (TG) or both or a degree of lower high-density lipoproteins.^14^ Various study shows that increased plasma triglyceride and LDL-c levels and a decreased concentration of HDL-c, as a significant risk factor for peripheral vascular diseases, stroke, and CAD.^15^

A study done revealed a significant association of all lipid levels, with the increased risk of ischemic stroke.^16^ Similarly, a randomised controlled trial carried among ischemic stroke patients in various part of world represented elevated baselines TG/HDL, and TC/HDL ratios as significant predictors for the future vascular risk.^17^ Similar one study show hypertriglyceridemia is among the greatest contributors to dyslipidemia which was similar to our study in which 58.3% patient had increased triglyceride.^18^

Gender-wise distribution showed that the stroke patients with dyslipidemia included 60% males and 40% females. Similarly, TC 40 (55.6%), and 33 (45.8%) LDL-c were high in male stroke patients whereas 33 (68.3%) females had high TG. Likewise, 34 (47.2%) males and 17 (35.4%) females had low HDL which shows various result in different studies.^7,8,13^

The prevalence of hypertension 61.9% in our patients. This finding is consistent with the various studies done in Nepal reported that 60% and 61.2% prevalence of hypertension respectively.^19,20^ The prevalence of hypertension in the South Asian population has been reported to be about 30% and is also reported to be significantly higher compared to the White population.^21^ The second most common CVD risk factor found in our patient was smoking as 58.3% of the patients reported as current smokers. This is similar to the previous studies in Nepalese and south-east population.^22,23^

The prevalence of smoking is higher in our study as smoking is considered as an acceptable behaviour for men but not for women in our society.^24^ In stroke patients of all ages, the highest prevalence of smoking is reported in Europe (28.7%) and in Southeast Asia (24.8%).^25^ History of alcohol consumption was present in 54% of the study participants in our study which is another common risk factor for ischemic stroke. This figure is higher compared to the previous report in Nepalese population.^18^’^23^ In all continents, alcohol consumption was associated with an increased risk of ischaemic stroke in general as stated in a study done.^25^ Other common risk factors of stroke in our population were type 2 diabetes (26%). Atrial fibrillation and positive family history of stroke were found in 6% each of the total population.

Ischemic stroke is multifactorial which requires a various approach. So early screening, monitoring and intervention of the different risk factors as well as awareness will help in combating the burden of the disease as its one of the leading causes of morbidity and disability in Asian population.^3^

The mean age of ischemic stroke patients with dyslipidemia in our study was 59.98 ±13.50 years with a range of 29-82 years. About 31.7% of patients belonged to less than 50 years (young stroke/nonelderly). The mean age reported by various studies were 60.22, 61 years and 61.7±14.9 years respectively which were close similar to our finding.^19,22,26^ The mean age of stroke in Western countries (69 years in USA and 71 years in Italy) was reported to be between 76-80 years.^4^ However, the low mean age of stroke patient in our study reflects demographic feature of this region. This may create awareness and knowledge about the modifiable cardiovascular risk factors in Nepal. This may also indicate that modifiable risk factors are not diagnosed and intervened timely.

Out of 120 stroke patients with dyslipidemia in our study, 60% were male and 40% were female which clearly shows male preponderance of stroke. This result was consistent with the finding from a study.^27^ Another study done in eastern Nepal also showed that the proportion of male patient was more than female patient (i.e., 69.3% and 30.7%).^23,27^ Several studies demonstrated that the incidence of stroke follows the global trend of male preponderance.^28^ Male sex may be a stronger risk factor for stroke. The clinical symptoms seen our study, such as hemiparesis, slurring of speech and aphasia, headache, vomiting, sensory disturbances and other were similar with the various study done by in India.^20,29,30^

The findings from our study cannot reflect the general population because it was a single-centre study with a small sample size. This was a descriptive crosssectional study, so a comparison and association with risk factors could not be established.

## CONCLUSIONS

The prevalence of dyslipidemia among ischemic stroke patients is higher than the similar studies done in similar settings. In our study, hypertriglyceridemia was the most common finding and hypertension was also found to be common. Similarly, awareness and knowledge about the modifiable CVD risk factors in Nepal is low. Possessing knowledge on the risk factors in each population can inform policy makers and healthcare planners on which factors should be targeted and what appropriate preventive measures should be taken to reduce the modifiable risk factor in our community level.
